# 肺鳞癌靶向治疗研究现状

**DOI:** 10.3779/j.issn.1009-3419.2013.10.11

**Published:** 2013-10-20

**Affiliations:** 1 200025 上海，上海交通大学医学院 Shanghai Jiao Tong University School of Medicine, Shanghai 200025, China; 2 200030 上海，上海市肺部肿瘤临床医学中心，上海交通大学附属胸科医院 Shanghai Lung Tumour Clinical Medical Center, Shanghai Chest Hospital Affiliated to Shanghai Jiao Tong University, Shanghai 200030, China

**Keywords:** 肺肿瘤, 鳞癌, 靶向治疗, Lung neoplasms, Squamous cell carcinoma, Targeted therapy

## Abstract

随着肺癌驱动基因研究的逐步深入，肺癌靶向治疗已取得较大进展。与肺腺癌相比，肺鳞癌的靶向治疗进展明显滞后。对肺腺癌临床疗效确切的靶向药物，如：表皮生长因子受体-酪氨酸激酶抑制剂（epidermal growth factor receptor-tyrosine kinase inhibitor, EGFR-TKI）、棘皮类微管相关样蛋白-4（echinodern microtubule-associated-protein-like 4, EML4）-间变型淋巴瘤激酶（anaplastic lymphoma kinase, ALK）融合基因抑制剂等，均对肺鳞癌疗效欠佳，目前肺鳞癌缺少有效的靶向治疗药物。因此，迫切需要对肺鳞癌的驱动基因和靶向治疗进行更深入的研究。本文将对肺鳞癌靶向治疗的研究现状作一综述。

## Abstract (en)

With the research of driver mutations, targeted therapy for lung cancer has made a great progress. Compared with lung adenocarcinoma, treatment of squamous cell lung cancer (SCC) has been far behind. While targeted therapies have improved outcomes for patients with lung adenocarcinoma, such as EGFR-TKIs, EML4-ALK inhibitors, molecularly targeted drugs are poorly active for SCC. SCC currently lacks therapeutically exploitable genetic alterations. These observations emphasize the need for new driver mutations and "druggable" targets in SCC patients. Combining with the research in recent years, this review will discuss the research status in targeted therapy for SCC.

肺癌死亡率居全球恶性肿瘤之首，在我国肺癌发病率呈现逐年上升趋势，年平均增长1.63%。继表皮生长因子受体酪氨酸激酶抑制剂（epidermal growth factor receptor tyrosine kinase inhibitor, EGFR-TKI）、抗EGFR单克隆抗体、肿瘤血管生成抑制药之后，克唑替尼（crizotinib）——靶向ALK和间叶组织上皮样变（mesenchymal-epithelial transition, MET）的小分子TKI，在肺腺癌的靶向治疗中显示出较好的临床疗效，但这些药物均对肺鳞癌疗效欠佳。

近年来，二代测序技术（Deep Sequence）用于肺鳞癌的研究，通过对肺鳞癌组织的全基因组和全外显子测序，纤维母细胞生长因子受体1（fibroblast growth factor receptor 1, FGFR1）扩增、盘状结构域受体2（discoidin domain receptor2, DDR2）突变、磷脂酰肌醇3-激酶（phosphatidylinositol 3-kinase, PI3K）通路改变、大鼠Kelch样ECH相关蛋白1（Kelch-like ECH-associated protein 1, KEAP1）和小鼠核因子E2相关因子2（nuclear factor erythroid-derived 2-like 2, NFE2L2）突变、SOX2扩增和TP63扩增等肺鳞癌驱动基因被陆续发现，为肺鳞癌的靶向治疗开创了新纪元。肺鳞癌的潜在靶点见图 1。

**1:**
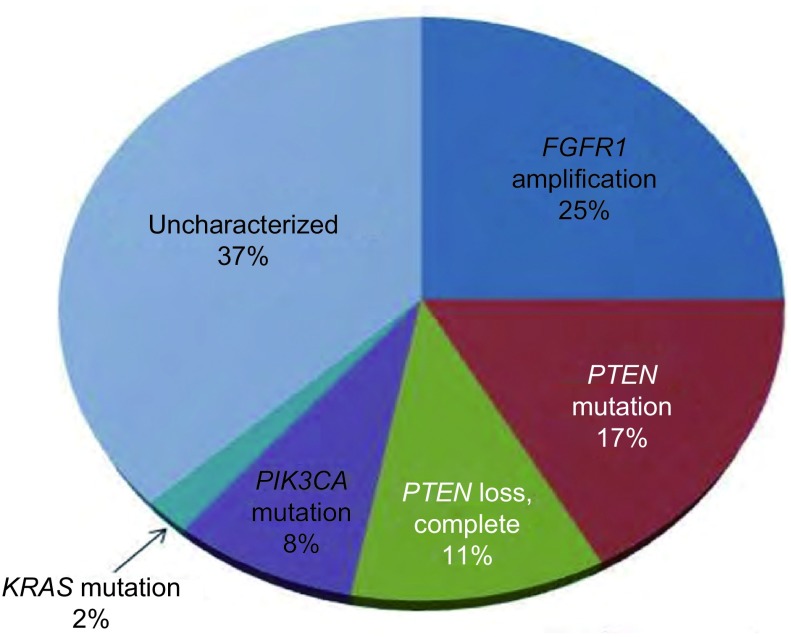
肺鳞癌的分子分型与驱动基因分析^35^ Molecular analysis of squamous cell lung cancer

## 1 *FGFR1*基因扩增

FGF/FGFR信号通路在胚胎发育、组织稳态、组织修复、伤口愈合和炎症等生理过程中发挥重要作用。FGFR与FGF配体结合，该家族有5个成员，其中FGFR1-4是单次跨膜的酪氨酸激酶受体。FGF配体家族有18个成员，分为激素样FGF（如FGF19, 21, 23）和经典的FGF（如FGF1-10, 16-18, 20），其中FGFR配体1缺乏酪氨酸激酶结构域。FGFs生物学功能广泛，包括营养神经、血管生成、诱导干细胞分化、组织修复和成骨。

FGFR1扩增是肺鳞癌的标志性改变之一。Weiss等^[1]^通过对155例原发肺鳞癌样本进行单核苷酸多态性（single nucleotide polymorphism, SNP）分析，发现染色体8p12位点的扩增很重要，并借助荧光原位杂交（fluorescence *in situ* hybridization, FISH）的方法检测了153例肺鳞癌样本，得出FGFR1的扩增频率为22%。Dutt等^[2]^用SNP微阵列分析了57例肺鳞癌样本，发现21%的肺鳞癌有FGFR1扩增，而腺癌仅为3%。这两项研究都证实，具有FGFR1扩增的细胞系，细胞的生长依赖于FGFR1介导的信号通路。

Weiss等^[1]^在异种移植FGFR1扩增肺癌细胞系的小鼠模型中观察到，使用FGFR抑制剂——PD173074治疗后肿瘤消退。一例FGFR1扩增的肺癌患者经过治疗，8周时靶病灶缩小33%，12周时同样观察到病灶缩小^[3]^。关于选择性FGFR1 TKI（AZD4547, BGJ398）的早期临床研究目前还在进行中。FGFR1扩增有望成为肺鳞癌新的治疗靶点。

## 2 *DDR2*突变

DDR是一种可以和胶原蛋白结合的受体酪氨酸激酶（receptor tyrosine kinase, RTK），可促进细胞的迁移、增殖和存活。在非小细胞肺癌（non-small cell lung cancer, NSCLC）尤其是肺鳞癌中，DDR1的上调与患者的无病生存期和总生存期（overall survival, OS）提高有关^[4]^。*DDR1*和*DDR2*突变见于不同类型的恶性肿瘤，DDR1、DDR2激酶结构域和非激酶结构域的突变均见于NSCLC^[5]^。2011年，Hammerman等^[6]^对290例肺鳞癌组织和细胞系进行了测序分析，证实*DDR2*的突变率为3.8%。

达沙替尼（dasatinib）是一个抗DDR1和DDR2的多靶点激酶抑制剂，尤其对具有*DDR2*突变的肿瘤有效。一项早期临床研究报道，1例*EGFR*野生型的肺鳞癌患者在接受达沙替尼和厄洛替尼联合治疗后，获得长达14个月的稳定期，但因毒性无法耐受而中断治疗。测序显示该患者DDR2激酶结构域为S768R突变。

DDR2抑制剂包括达沙替尼、尼洛替尼（nilotinib）和伊马替尼（imatinib），食品药物管理局（Food and Drug Administration, FDA）已批准这三个药物用于慢性粒细胞性白血病（chronic myelogenous leukemia, CML）的治疗。此外，达沙替尼还是靶向Bcr-Abl和Src激酶的小分子抑制剂，一项达沙替尼联合厄洛替尼治疗NSCLC的随机队列研究正在进行中。

## 3 PI3K/蛋白激酶B（PKB或AKT）/哺乳动物雷帕霉素靶蛋白（mammalian target of rapamycin, mTOR）通路

PI3K是一个异源二聚体，由P85调节亚基和P110催化亚基组成，可以将磷脂酰肌醇磷酸氢盐磷酸化为磷脂酰肌醇三磷酸盐。PTK信号可以活化PI3K，如EGFR、胰岛素样生长因子1受体（insulin-like growth factor receptor, IGF1-R）和人类表皮生长因子受体2（human epidermal growth factor receptor 2, HER-2/neu）可以使PI3K活化^[7]^。PI3K信号转导通路对细胞的存活、代谢、运动和血管发生极为重要。相比肺腺癌，肺鳞癌中更常见PI3K/类脂磷酸酶（phosphatase and tensin homology deleted on chromosome ten, PTEN）/AKT/mTOR通路的异常。

磷酸化磷脂酰肌醇催化亚单位A抗体（PIK3CA）编码PI3Ks的p110催化亚单位，即PI3Kp110α。*PIK3CA*基因突变见于具有*EGFR*突变的肿瘤，在肺鳞癌和腺癌中同样常见^[8]^。肺鳞癌中*PIK3CA*的突变率为3.6%-6.5%^[8]^，PIK3CA扩增见于男性、吸烟的肺鳞癌患者^[9]^。PIK3CA突变集中在两个区域——9号和20号外显子，二者分别编码蛋白的螺旋结构域和激酶结构域。突变导致脂质激酶活性增强，以及PI3K/AKT信号通路的活化。Okudela等^[9]^用FISH法测得43%的日本肺鳞癌患者基因拷贝数增加，Ji等^[10]^用聚合酶链反应（polymerase chain reaction, PCR）法记录到42%的中国肺鳞癌患者有PIK3CA扩增。这些结果均与肺鳞癌的比较基因组杂交（comparative genomic hybridization, CGH）研究一致。

PI3K下游基因*AKT1*的*E17K*突变导致AKT1的活化^[11]^。5.6%（2/36）的肺鳞癌有*E17K*突变，肺腺癌中*E17K*突变相对罕见^[12]^。

*PTEN*是一个负性调节PI3K/AKT/mTOR轴的抑癌基因，PTEN缺失导致该通路的活性增强。Soria等^[13]^用免疫组织化学（immunohistochemistry, IHC）方法测得，肺鳞癌中PTEN表达缺失和PTEN甲基化分别占24%和35%。Jin等^[14]^报道*PTEN*的突变率，肺鳞癌为10.2%、肺腺癌1.7%。

遗传积累和肿瘤生物学研究表明，PI3K通路对肿瘤细胞的生长和生存作用明显。临床中靶向PI3K/AKT通路的抑制剂有：PI3K/mTOR抑制剂、PI3K抑制剂、AKT抑制剂和mTOR抑制剂^[15]^。靶向PI3K和mTOR的小分子抑制剂BEZ235（Novartis, Basel, Switzerland）已经在小鼠实验中显示出抗肿瘤活性^[16]^。PI3K抑制剂仍在早期临床研发阶段，但单一抑制剂的有效率很低^[17]^。

目前正在各种实体瘤中评估PI3K通路抑制剂的疗效，包括PI3K的不同亚型抑制剂、AKT1和mTOR抑制剂、以及PI3K/mTOR抑制剂。这一研究的意义在于揭示了*PIK3CA*突变和其他癌基因（如*KRAS*、*BRAF*和*EGFR*，以及肺腺癌中的*EML4-ALK*）的畸变共存^[18]^。而肺鳞癌中*PIK3CA*突变和其他癌基因畸变的共存程度仍有待观察，这将决定靶向这些位点的联合治疗是否有效。

## 4 KEAP1和NFE2L2

KEAP1和NFE2L2相互结合，调节细胞的氧化损伤应答和异生物质应激，在很多肿瘤中发现了KEAP1和NFE2L2的改变。无应激条件下，CUL3-KEAP1泛素连接酶E3复合体使NFE2L2通过泛素化途径降解。在应激条件下，KEAP1的半胱氨酸残基被修饰，使得NFE2L2泛素化以及稳定性下降。KEAP1是细胞的亲电子感受器。NFE2L2是转录激活剂，参与细胞保护基因的表达、谷胱甘肽的合成、活性氧清除、异生物质代谢以及药物运输。IHC方法证实NFE2L2的表达增加和KEAP1的表达下降与NSCLC OS缩短有关^[19]^。

遗传和表观遗传机制都会使KEAP1-NFE2L2通路失调。NSCLC中有*KEAP1*和*NFE2L2*基因突变。*KEAP1*的突变失活最初在肺癌细胞系中报道，该突变失活见于19%的NSCLC，且多为肺腺癌^[20]^。而NFE2L2的编码区突变主要见于肺鳞癌，并和既往吸烟有关。Shibata等^[21]^报道在原发性NSCLC（多为肺鳞癌）和25%的头颈部肿瘤中，*NFE2L2*的点突变率为10.7%。一项研究^[22]^报道，*NFE2L2*突变的细胞中有mTOR的激活。Kim等^[23]^证实，NSCLC中*NFE2L2*的突变率为8%，在皮肤鳞癌和食管鳞癌中也存在*NFE2L2*突变。目前尚无针对NFE2L2的特异性抑制剂。

## 5 SOX2扩增

在肺鳞癌和食管癌中，*SOX2*转录基因位于染色体3q26.33位点。SOX2参与食道和气管发育，在成熟细胞重编程、成为干细胞的过程中发挥了重要作用^[24]^。运用CGH方法在3q26-qter位点寻找基因靶点时首次鉴定了*SOX2*基因。3q26-qter在60%-80%的不同类型鳞癌中都有扩增，在20%的肿瘤中为高水平扩增^[24-26]^。

Wilbertz等^[27]^对患有NSCLC的两个独立队列进行研究，以观察SOX2扩增在其中发挥的作用。由IHC方法得出，肺鳞癌中SOX2的平均表达明显高于肺腺癌（*P* < 0.001）。FISH法得出在68%的肺鳞癌中，SOX2低水平扩增，肺腺癌仅6%，8%的肺鳞癌SOX2高水平扩增。IHC方法得出，SOX2高表达和OS增加有关（*P*=0.036）；而SOX2高水平扩增，OS改善并不明显（*P*=0.078）^[27]^。

SOX2是一个既控制胚胎干细胞多能性，又调节气管支气管上皮形态发生的转录因子。SOX2在起源于正常上皮的侵袭性肿瘤的发生发展中发挥作用，并驱使鳞状组织学标记（如P63）表达，这一假设较为认可。仅有SOX2扩增尚不足以引起恶性转化，这一过程可能需要其他致癌因素的参与。尽管SOX2扩增尚未成为治疗靶点，但从肿瘤过表达细胞周期蛋白D1（cyclinD1）的角度分析，抑制细胞周期或许可以成为一种新的治疗手段^[28]^。

## 6 TP63扩增

P63是一个转录因子，反式激活*P53*靶基因，目前认为P63是鳞状上皮基底细胞的重要干细胞因子、也是鳞癌发生中的关键癌基因。TP63扩增的鳞状上皮和肺癌中通常见到截短的P63α剪接变体的表达^[29]^。肺鳞癌中有TP63扩增和TP63过表达。Massion等^[30]^用FISH法得出88%的肺鳞癌有TP63扩增。虽然TP63的扩增和过表达之间没有相关性，但是IHC方法证实，*TP63*基因组的扩增和过表达与肺鳞癌的生存改善相关^[30]^。

## 7 *EGFR*和*KRAS*突变、EML4-ALK重排

肺癌靶向治疗取得成功，很大程度上取决于驱动基因的发现。EGFR和EML4-ALK的小分子抑制剂能明显改善肺腺癌的缓解率和无进展生存期（progression free survival, PFS）。

*EGFR*突变最常见于19号外显子缺失和21号外显子点突变（L858R）。EGFR v Ⅲ突变以胞外段2-7号外显子267个氨基酸缺失为特征。这一区域编码受体的胞外结构域部分，EGFR v Ⅲ缺失导致配体二聚化和磷酸化。EGFR v Ⅲ的剪切变异，使EGFR获得自身磷酸化的能力，在无配体存在的情况下激活其下游的丝裂原活化蛋白激酶（mitogen activated protein kinase, MAPK）、细胞外调节蛋白激酶（extracellular regulated protein kinase, ERK）等通路，导致肿瘤的发生发展。EGFR v Ⅲ突变常见于高级别神经胶质瘤和头颈鳞癌。直接测序得出肺鳞癌的发病率为5%-8%^[31, 32]^。EGFR v Ⅲ突变对可逆性TKI治疗不敏感。

*KRAS*突变是高加索人群NSCLC中最常见的致癌突变，见于约25%的腺癌。肺癌中*KRAS*突变主要位于12和13号密码子。*KRAS*突变阳性患者不仅对化疗敏感性降低，且通常对EGFR-TKI治疗不敏感。尽管目前尚未研发出直接靶向*KRAS*突变的药物，但利用新一代的靶向治疗联合化疗，或联合PI3K及MEK抑制剂等靶向治疗策略正在研发中。

*EGFR*野生型和*KRAS*野生型的肿瘤常发生EML4-ALK易位^[33]^。ALK可以通过激活RAS-MEK-ERK、JAK3-STAT3和PI3K-AKT信号通路使细胞增殖。对ALK抑制剂克唑替尼的大型Ⅰ期临床研究证实，肿瘤患者（含ALK易位）整体反应率为57%，疾病控制率为90%。更有效的ALK抑制剂和靶向获得性耐药的治疗策略仍在研发中。

## 8 总结

*DDR2*突变和FGFR1扩增导致跨膜受体对应的下游信号增加。PIK3CA、PTEN和AKT畸变导致PI3K通路活性增加。*KEAP1*或*NFE2L2*突变导致NFE2L2介导的细胞保护基因的表达增加。SOX2扩增可能会导致SOX2介导的基因活化，这些基因控制多能性以及肿瘤的生长。

癌症基因组图谱研究网络组（The Cancer Genome Atlas Research Network, TCGA）报道了一项178例肺鳞癌的组织病理学研究，该研究基于DNA拷贝数、体细胞外显子突变、mRNA测序、mRNA表达和启动子甲基化。通过分析178个癌症患者的基因组，TCGA得出96%（171/178）的患者基因组中至少存在一个突变位点，突变位点位于酪氨酸激酶、丝氨酸-苏氨酸激酶、PI3K催化亚基和调节亚基、核激素受体、G蛋白耦联受体以及酪氨酸磷酸酯酶区域。除外本综述中讨论的肺鳞癌中存在的异常基因，突变基因中值得关注的有*TP53*、*CDKN2A*、*MLL2*、*NOTCH1*、*RB1*和*HLA-A*。下述通路值得关注，CDKN2A/RB1，NFE2L2/KEAP1/CUL3，PI3K/AKT和SOX2/TP63/NOTCH1。这些改变为细胞周期调控、氧化应激反应、凋亡信号和鳞状细胞分化的异常提供了证据^[34, 35]^。肺腺癌通常为单个驱动基因改变，而肺鳞癌的复杂之处表现为数个驱动基因或几条信号通路同时变化，提示联合靶向治疗可能对肺鳞癌的治疗更有效。

## References

[1] Weiss J, Sos ML, Seidel D (2010). Frequent and focal FGFR1 amplification associates with therapeutically tractable FGFR1 dependency in squamous cell lung cancer. Sci Transl Med.

[2] Dutt A, Ramos AH, Hammerman PS (2011). Inhibitor-sensitive FGFR1 amplification in human non-small cell lung cancer. PLoS One.

[b3] 3Wolf J. A phase Ⅰ dose escalation study of NVP-BGJ398, a selective pan FGFR inhibitor in genetically preselected advanced solid tumors [abstract]. In: Proceedings of the 103^rd^ Annual Meeting of the Ameri-can Association for Cancer Research; 2012 Mar 31-Apr 4; Chicago, IL. Philadelphia (PA) : AACR; 2012. Abstract nr LB-122.

[4] Ford CE, Lau SK, Zhu CQ (2007). Expression and mutation analysis of the discoidin domain receptors 1 and 2 in non-small cell lung carcinoma. Br J Cancer.

[5] Davies H, Hunter C, Smith R (2005). Somatic mutations of the protein kinase gene family in human lung cancer. Cancer Res.

[6] Hammerman PS, Sos ML, Ramos AH (2011). Mutations in the *DDR2* kinase gene identify a novel therapeutic target in squamous cell lung cancer. Cancer Discov.

[7] Zito CR, Jilaveanu LB, Anagnostou V (2012). Multi-level targeting of the phosphatidylinositol-3-kinase pathway in non-small cell lung cancer cells. PLoS One.

[8] Kawano O, Sasaki H, Endo K (2006). *PIK3CA* mutation status in Japanese lung cancer patients. Lung Cancer.

[9] Okudela K, Suzuki M, Kageyama S (2007). *PIK3CA* mutation and amplification in human lung cancer. Pathol Int.

[10] Ji M, Guan H, Gao C (2011). Highly frequent promoter methylation and PIK3CA amplification in non-small cell lung cancer (NSCLC). BMC Cancer.

[11] Do H, Solomon B, Mitchell PL (2008). Detection of the transforming *AKT1* mutation E17K in non-small cell lung cancer by high resolution melting. BMC Res Notes.

[12] Malanga D, Scrima M, De Marco C (2008). Activating *E17K* mutation in the gene encoding the protein kinase AKT1 in a subset of squamous cell carcinoma of the lung. Cell Cycle.

[13] Soria JC, Lee HY, Lee JI (2002). Lack of PTEN expression in non-small cell lung cancer could be related to promoter methylation. Clin Cancer Res.

[14] Jin G, Kim MJ, Jeon HS (2010). *PTEN* mutations and relationship to *EGFR*, *ERBB2*, *KRAS*, and *TP53* mutations in non-small cell lung cancers. Lung Cancer.

[15] Engelman JA (2009). Targeting PI3K signalling in cancer: opportunities, challenges and limitations. Nat Rev Cancer.

[16] Engelman JA, Chen L, Tan X (2008). Effective use of PI3K and MEK inhibitors to treat mutant *Kras* G12D and *PIK3CA* H1047R murine lung cancers. Nat Med.

[b17] 17Shapiro G. Phase Ⅰ dose-escalation study of XL147, a PI3K inhibitor administered orally to patients with solid tumors. J Clin Oncol, 2009, 27: 15s (abstr 3500).

[18] Chaft JE, Arcila ME, Paik PK (2012). Coexistence of PIK3CA and other oncogene mutations in lung adenocarcinoma-rationale for comprehensive mutation profiling. Mol Cancer Ther.

[19] Solis LM, Behrens C, Dong W (2010). Nrf2 and Keap1 abnormalities in non-small cell lung carcinoma and association with clinicopathologic features. Clin Cancer Res.

[20] Singh A, Misra V, Thimmulappa R. K (2006). Dysfunctional KEAP1-NRF2 interaction in non-small-cell lung cancer. PLoS Med.

[21] Shibata T, Ohta T, Tong KI (2008). Cancer related mutations in NRF2 impair its recognition by Keap1-Cul3 E3 ligase and promote malignancy. Proc Natl Acad Sci U S A.

[22] Shibata T, Saito S, Kokubu A (2010). Global downstream pathway analysis reveals a dependence of oncogenic NF-E2-related factor 2 mutation on the mTOR growth signaling pathway. Cancer Res.

[23] Kim YR, Oh JE, Kim MS (2010). Oncogenic *NRF2* mutations in squamous cell carcinomas of oesophagus and skin. J Pathol.

[24] Bass AJ, Watanabe H, Mermel CH (2009). *SOX2* is an amplified lineage-survival oncogene in lung and esophageal squamous cell carcinomas. Nat Genet.

[25] Hussenet T, du Manoir S (2010). SOX2 in squamous cell carcinoma: amplifying a pleiotropic oncogene along carcinogenesis. Cell Cycle.

[26] Hussenet T, Dali S, Exinger J (2010). *SOX2* is an oncogene activated by recurrent 3q26.3 amplifications in human lung squamous cell carcinomas. PLoS One.

[27] Wilbertz T, Wagner P, Petersen K (2011). *SOX2* gene amplification and protein overexpression are associated with better outcome in squamous cell lung cancer. Mod Pathol.

[28] Lu Y, Futtner C, Rock JR (2010). Evidence that SOX2 overexpression is oncogenic in the lung. PLoS One.

[29] Hibi K, Trink B, Patturajan M (2000). *AIS* is an oncogene amplified in squamous cell carcinoma. Proc Natl Acad Sci U S A.

[30] Massion PP, Taflan PM, Jamshedur Rahman SM (2003). Significance of p63 amplification and overexpression in lung cancer development and prognosis. Cancer Res.

[31] Ji H, Zhao X, Yuza Y (2006). Epidermal growth factor receptor variant Ⅲ mutations in lung tumorigenesis and sensitivity to tyrosine kinase inhibitors. Proc Natl Acad Sci U S A.

[32] Sasaki H, Kawano O, Endo K (2007). EGFR v Ⅲ mutation in lung cancer correlates with increased EGFR copy number. Oncol Rep.

[33] Horn L, Pao W (2009). EML4-ALK: honing in on a new target in non-small-cell lung cancer. J Clin Oncol.

[34] The Cancer Genome Atlas Research Network (2012). Comprehensive genomic characterization of squamous cell lung cancers. Nature.

[35] Paik PK, Hasanovic A, Wang L (2012). Multiplex testing for driver mutations in squamous cell carcinomas of the lung. J Clin Oncol.

